# PI3K/Akt/mTOR signaling pathway and targeted therapy for glioblastoma

**DOI:** 10.18632/oncotarget.7961

**Published:** 2016-03-07

**Authors:** Xiaoman Li, Changjing Wu, Nianci Chen, Huadi Gu, Allen Yen, Liu Cao, Enhua Wang, Liang Wang

**Affiliations:** ^1^ Key Laboratory of Medical Cell Biology, Ministry of Education, China Medical University, Shenyang, China; ^2^ Class 9 of the 97th Clinical Medicine of Seven-Year Program, China Medical University, Shenyang, China; ^3^ Department of Pathology, The College of Basic Medical Sciences, China Medical University, Shenyang, China; ^4^ School of Medicine, The University of Texas Southwestern Medical Center, Dallas, Texas, United States of America; ^5^ Department of Pathology, The First Affiliated Hospital of China Medical University, Shenyang, China

**Keywords:** glioblastoma, EGFR, PI3K/Akt/mTOR pathway, targeted therapy

## Abstract

Glioblastoma multiform (GBM) is the most common malignant glioma of all the brain tumors and currently effective treatment options are still lacking. GBM is frequently accompanied with overexpression and/or mutation of epidermal growth factor receptor (EGFR), which subsequently leads to activation of many downstream signal pathways such as phosphatidylinositol 3-kinase (PI3K)/Akt/rapamycin-sensitive mTOR-complex (mTOR) pathway. Here we explored the reason why inhibition of the pathway may serve as a compelling therapeutic target for the disease, and provided an update data of EFGR and PI3K/Akt/mTOR inhibitors in clinical trials.

## INTRODUCTION

### Glioblastoma multiform

Glioblastoma multiform (GBM), WHO grade IV, is the most common and aggressive glioma of all primary brain tumors, exhibiting a high rate of recurrence and poor prognosis due to the invasive nature of the tumor [1, 2]. Considering the location and diffusely infiltrating nature of the tumor, complete surgical resections are challenging. Standard therapy for GBM is radiation plus the chemopeutic agent temozolomide (TMZ). The cytotoxicity of TMZ is thought to be primarily due to alkylation of DNA hence leading to DNA damage and tumor cell death [3]. However, the activation of PI3K/Akt/mTOR pathway leads to the development of drug resistance thereby dampening the therapeutic effect of TMZ [4]. The five year survival rate for glioblastoma is less than 5% in adults [5-7]. The occurrence of GBM is frequently associated with molecular changes in epidermal growth factor receptor (EGFR) and phosphatidylinositol 3-kinase (PI3K)/Akt/rapamycin-sensitive mTOR-complex (mTOR) pathways. The frequency of genetic alterations such as overexpression EGFR, activating mutations of PI3CA (p110) or PIK3R1 (P85), or loss of PTEN expression has been estimated to around 88% [8-12]. GBM patients with an activated PI3K/Akt/mTOR pathway also have poor prognosis than patients without oncogenic activation of the pathway [13]. Therefore, inhibitors targeting EGFR and PI3K/Akt/mTOR pathway have emerged as potential treatment for GBM [14-18]. Currently, a series of inhibitors targeting EGFR and PI3K/Akt/mTOR pathway are evaluated in preclinical and clinical studies as single agent or in combination with the traditional treatment [19]. It is of particular interest to explore whether those inhibitors are effective to restore the therapeutic sensitivity.

### EGFR and PI3K/Akt/mTOR signal transduction pathway

EGFR is a type of receptor tyrosine kinases (RTKs), playing a central role in cell division, migration, adhesion, differentiation and apoptosis [20, 21]. EGFR comprises of extracellular ligand binding domain, transmembrane domain and intracellular tyrosine kinase domain. Upon binding to various of ligands, such as EGF and TGFα, EGFR is activated through homodimerization or heterdimerization on the cell surface and subsequently leads to the phosphorylation of its intracellular tyrosine kinase domain [22]. The activation of EGFR results in activation of multiple downstream signal transduction pathways such as PI3K/Akt/mTOR pathway [23].

Members of the PI3K family are lipid kinases involved in multiple cellular process, including proliferation, differentiation, migration, metabolism and survival [24]. PI3K is generally classified into three classes according to their substrate specificity and subsequence homology, among which, the class I is most vital to the tumorigenesis. Class I consisted of a catalytic subunit p110 (α, β, γ) and a regulator subunit p85. A fourth p110 isoform (p110δ) is paired with the p101 regulatory subunit in class IB PI3Ks. Upon ligand binding, phosphorylated tyrosine residing in activated RTKs will bind to p85. The subsequent conformation change will release the catalytic subunit p110 [25], where activated p110 phosphorylated the phosphatidy-linositol-3, 4-bisphosphate (PIP2) into the second messenger phosphatidylinositol-3, 4, 5-bisphosphate (PIP3). This reaction can be reversed by the PI3K antagonist PTEN (phosphatase and tensin homolog deleted on chromosome ten) [26]. Subsequently, PIP3 will recruit the downstream Akt to inner membranes and phosphorylates Akt on its serine/threonine kinasesites (Thr308 and Ser473) [27, 28]. Activated Akt is involved in the downstream mTORC1 mediated response to biogenesis of protein and ribosome.

**Figure 1:**
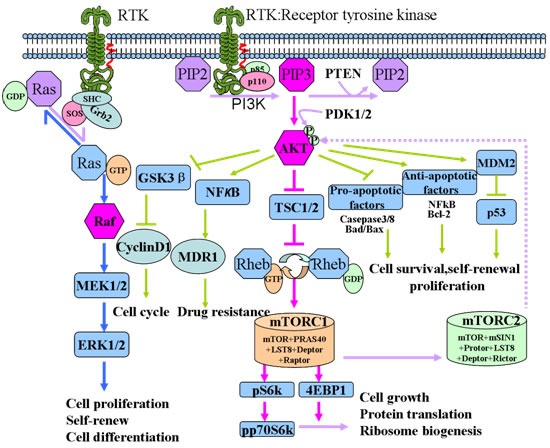
Schematic representation of the PI3K/Akt/mTOR signaling pathway Upon relevant ligand binding, RTK, such as EGFR, is activated and subsequently inducing a series of cascade reaction. First, the regulator subunits of PI3K, p85, dimerize and release its catalytic subunit p110. p110 enables the membrane protein PIP2 to phosphorylate into PIP3. PIP3 begins to recruit the downstream Akt to inner membranes and phosphorylated the serine/threonine kinase (Thr308 and Ser473) sites by phosphoinositide-dependent kinase 1/2 (PDK1/2). Activated Akt is involved in the downstream mTORC1 mediated response to biogenesis of protein and ribosome. Besides that, activated Akt is also involved in the regulation of cell cycle and pro-apoptotic and anti-apoptotic factors mediated choices of cell apoptosis and survival. Additionally, it is also involved in the NFκB/MDR1 mediated drug resistance.

In PI3K pathway, mTOR acts as both a downstream effector and an upstream regulator [29, 30]. mTOR resides in rapamycin-sensitive mTOR-complex (mTORC1) and a rapamycin-insensitive complex (mTORC2) [31, 32]. The activated Akt inhibits tuberous sclerosis complex (TSC) 1/2 activity, thereby initiate the mTORC1-mediate signaling pathway, involving in the phosphorylation of ribosomal protein S6 kinase (pS6k), eukaryotic initiation factor 4E (eIF4E) and eukaryotic initiation factor binding protein 1(4EBP1), which participate in protein translation, ribosome biogenesis as well as cell growth [33, 34]. The mTORC2 phosphorylates Akt at Ser-473, and then further takes part in cell survival, metabolism, proliferation, and cytoskeletal organization [31, 35]. Within PI3K signaling pathway, another important molecule is PTEN. As clinical research revealed, the EGFR or PTEN mutation would lead to continuous activation of PI3K/Akt/mTOR signaling pathway, thereby contributing to the tumorigenesis and cancer therapy resistance (Figure 1).

## THE DEVELOPMENT OF TARGETED THERAPY TOWARDS EGFR AND PI3K/AKT/MTOR

### EGFR inhibitors

EGFR alteration, including overexpression or gene amplification, is the most frequent form of genetic mutation, occurred in 40-50% of glioblastomas [36, 37]. Logically, EGFR is a promising target for the treatment of GBM. Though promising results was shown in preclinical data, targeting EGFR in clinical trials revealed marginal effects. An overview of ongoing clinical trial in GBM is summarized in Table 1. Information about clinical trials has been retrieved from www.clinicaltrials.gov. In the clinical trial NCT00250887, the effectiveness of EGFR tyrosine kinase inhibitor Gefitinib was tested in recurrent glioblastoma. Though EGFR was successfully -dephosphorylated, the downstream target remains constitutively active. Therefore the effectiveness was unsatisfactory [38]. Erlotinib, another selective EGFR inhibitor, also showed minimal effect to treat the recurrent glioblastoma (NCT00086879) [39]. Other than single agent treatment, combination therapy was also explored. When Erlotinib was combined with TMZ and radiotherapy in a phase I/II trial, no sign of benefit was showed compared with TMZ controls (NCT00039494) [40, 41]. Additionally, the combination therapy of Erlotinib with VEGF antibody also shows no obvious survival benefit [42]. The failure of targeting EGFR is generally due to the hyperactivation of downstream PI3K/Akt signaling, so the downstream components represents an attractive target for the treatment of malignant brain tumors.

**Table 1:** Ongoing clinical trials in brain tumors targeting EGFR

Drug	Targets	Combination Partner	Patient group	Phase	State	Trail ID
Gefitinib	EGFR		recurrent glioblastoma	II	completed	NCT00250887
	EGFR		GBM	II	completed	NCT00014170
	EGFR		GBM	II	completed	NCT00016991
	EGFR		brain and central nervous system tumors	II	completed	NCT00025675
	EGFR	radiation	GBM	I/II	completed	NCT00052208
	EGFR	radiation	GBM	II	completed	NCT00238797
Gefitinib, Temozolomide	EGFR		brain and central nervous system tumors	I	completed	NCT00027625
Gefitinib, Irinotecan	EGFR, topoismerase I		refractory solid tumor	I	completed	NCT00132158
Erlotinib	EGFR		GBM	II	completed	NCT00337883
	EGFR		GBM	II	unknown	NCT00054496
	EGFR		GBM and other brain tumors	I/II	ongoing	NCT00045110
	EGFR		GBM	I/II	completed	NCT00301418
	EGFR	radiation	brain/central nervous system tumors	I/II	completed	NCT00124657
	EGFR	cytoreductive surgery	recurrent malignant gliomas		ongoing	NCT01257594
Erlotinib, Temozolomide	EGFR	radiation	GBM, gliosarcoma	II	completed	NCT00187486
	EGFR	radiation	GBM	II	completed	NCT00274833
	EGFR	radiation	GBM	II	completed	NCT00039494
Erlotinib, Temozolomide, Carmustine	EGFR		glioblastoma, gliosarcoma	II	completed	NCT00086879
Erlotinib, Bevacizumab.	EGFR, VEGF		glioblastoma, gliosarcoma	II	completed	NCT00671970
Erlotinib, Bevacizumab, Temozolomide	EGFR, VEGF	radiation	GBM	II	ongoing	NCT00720356
Erlotinib, Sorafenib	EGFR, RAF, VEGFR		GBM	II	completed	NCT00445588
Erlotinib, Dasatinib	EGFR, SRC		GBM	I	completed	NCT00609999
Dacomitinib	EGFR		recurrent glioblstoma	II	ongoing	NCT01520870
Afatinib	EGFR		refractory solid tumors	II	completed	NCT00875433
AEE788	EGFR		GBM	I/II	completed	NCT00116376
Lapatinib	EGFR		GBM	I/II	completed	NCT00099060
Lapatinib	EGFR		malignant brain tumors	II	completed	NCT00107003
Nimotuzumab	EGFR		GBM		completed	NCT00561873
Nimotuzumab, Temozolomide	EGFR	radiation	GBM	III	completed	NCT00753246
EGFR Bi-armed Autologous T cells	EGFR, CD3		glioblastoma, gliosarcoma recurrent neoplasm	I/II	not yet recruiting	NCT02521090
AMG 595	EGFR		GBM	I	ongoing	NCT01475006
Sym004	EGFR		recurrent glioblastoma	II	ongoing	NCT02540161
Cetuximab, Temozolomide	EGFR	radiation	GBM	I/II	unknown	NCT00311857
Cetuximab, Bevacizumab, Irinotecan	EGFR, VEGF, topoismerase I		GBM	II	completed	NCT00463073
Afatinib, Temozolomide	EGFR	radiation	GBM	I	ongoing	NCT00977431
Afatinib, Temozolomide	EGFR		GBM	II	ongoing	NCT00727506

Clinical data related to the EGFR was searched until Nov, 2015.

### PI3K inhibitors

Currently, the PI3K inhibitors as a single agent or combined with other therapies are being tested in a number of clinical trials (Table 2) [43]. There are pan-PI3K inhibitors and isoform specific PI3K inhibitors [14]. The first generation of pan-PI3K inhibitors is represented by wortmannin and LY294002 [44, 45]. They have showed anti-cancer effect *in vivo* and *in vitro* [46-49]. However, both drugs were halted at preclinical studies due to the toxicity, poor pharmacodynamics and selectivity. A new generation of PI3K inhibitors, BKM120 and PX-866, exhibit better drug properties such as high stability and low side effects [50, 51]. BKM120 has anti-proliferative and pro-apoptotic activity in a number of tumor cell lines, human tumor xenograft models and cancer patients bearing PI3K activating mutations [52]. BKM120 was smoothly passed phase I clinical trial and now is undergoing phase II trial among patients with recurrent glioblastoma and activated PI3K pathway (NCT01339052) (Table 2) [50]. At present, BKM120 is also undergoing several clinical trials in combination with radiation (NCT01473901), anti-VEGF monoclonal antibody Bevacizumab (NCT01349660), LDE225 (NCT01576666) and INC280 (NCT01870726) [53]. PX-866 could bind with the catalytic domain of ATP and it acts as an irreversible inhibitor. Though PX-866 could increase median survival time of the animals and show significant anti-tumor activity in GBM xenograft models [54, 55], the recent completed clinical study showed the overall response rate was low (NCT01259869) [56].

**Table 2:** Ongoing clinical trials in brain tumors targeting PI3K

Drug	Targets	Combination partner	Patient group	Phase	State	Trail ID
BKM120	Pan-PI3K	surgery	recurrent glioblastoma	II	ongoing,	NCT01339052
		Bevacizumab	relapsed/refractory GBM	I/II	recruiting	NCT01349660
		LDE225	advanced solid tumor	I	completed	NCT01576666
		INC280	recurrent glioblastoma	I/II	recruiting	NCT01870726
BKM120, Temozolomide	Pan-PI3K	radiation	glioblastoma	I	ongoing	NCT01473901
PX-866	Pan-PI3K		GBM	II	completed	NCT01259869

Clinical data related to the EGFR was searched until Nov, 2015.

### Akt inhibitors

Akt is a central player in the EGFR/PI3K signaling pathways. Evidence shows that Akt play an important role in tumor proliferation and radiosensitivity [57]. One of the most promising Akt inhibitor, perifosine, inhibits Akt activity by preventing its translocation to the cell membrane [58, 59]. Currently, perifosine is being clinically tested in a number of different cancers [60, 61]. Perifosine has several drawbacks such as limited ability to penetrate blood-brain-barrier (BBB) and gastrointestinal side effects. A phase II trial of perifosine in recurrent GBM was ongoing but only marginal effect was shown (NCT00590954).

### mTOR inhibitors

As downstream targets of phosphorylated Akt, inhibition of mTOR would also be another therapeutic approach to reduce the effects of constitutively activate Akt in GBM. mTORC1 inhibitors mainly contain rapamycin (sirolimus) and its analogues, such as RAD001 (everolimus), CCL-779 (temsirolimus) and AP23573 (ridaforolimus) [62]. Rapamycin inactivate mTORC1 through altering the conformation of the kinase. Though rapamycin and its analogues exhibit efficacy of mTOR inhibitors in both *in vitro* and *in vivo* models [63, 64], they would arose hyperactivation of Akt and mTORC2 by some feedback loop and pathway crosstalk [65]. Rapamycin shows anti-tumor activity in a phase I trial for patients with recurrent PTEN-deficient glioblastoma (NCT00047073) [66]. Unfortunately, phase II clinical trials for rapamycin analogs fail to achieve promising results (NCT00515086, NCT00016328, NCT00022724, and NCT00087451) [67-71]. The limited efficacy might result from the feedback loops and crosstalk with other pathways. Recently, more exploration was focusing on the combination treatment of rapamycin analogs with other modalities [71]. The combination of EGFR inhibitor erlotinib with sirolimus or temsirolimus was tested in clinical trials (NCT00112736 and NCT0062243). However, either of trial shows promising results [72, 73]. A phase II study of everolimus with bevacizumab as part of first-line modality therapy for glioblastoma was feasible and efficacious (NCT00805961) [74], further studies are still need. As combined inhibition of Akt and mTOR by perfosine and temsirolimus inhibited murine glioblastoma growth no matter PTEN status, a phase I/II trial in recurrent high-grade gliomais ongoing (NCT01051557) [75, 76]. Metformin is a widely prescribed antidiabetic drug and many studies indicate that metformin inhibits cancer proliferation through the inhibition of mTOR [77]. The efficacy of metformin on glioblastoma was tested in clinical trial NCT01430351 and NCT02149459. In NCT02149459, metformin was combined with radiotherapy. In NCT01430351, metformin was combined with TMZ. Both of the trials are still in phase I state.geting specifically mTORC2 could thereby be a better approach, since it would directly block Akt phosphorylation without perturbing the mTORC1-dependent feedback loops [78, 79]. In contrast to mTORC1, mTORC1/2 inhibitors can restrain Akt phosphorylation at Ser473, thus also inhibit mTORC2 at the same time [63]. AZD8055 is a potent small molecular ATP-competitive inhibitor. *In vivo*, AZD-8055 reduced S6 and Akt phosphorylation thereby leading to the reduction of tumor growth [80]. It is implicated that AZD8055 may provide a more promising therapeutic strategy than rapamycin and analogues [81]. Currently AZD8055 has completed the phase I clinical trials (NCT01316809).

### PI3K/mTOR dual inhibitor

Since mTORC1 inhibitors could induce the loss of feedback inhibition of PI3K activation, drugs targeting PI3K and mTOR kinase simultaneously thereby become a superior option [82]. Active site ATP-competitor is a class of dual PI3K-mTOR inhibitor, which structurally targets the kinase domains of both PI3K and mTOR. PI-103 was the first dual mTOR/PI3K inhibitors that inhibited mTOR in an ATP-competitive manner [83]. *In vivo* study showed that PI-103 led to G0-G1 cell cycle arrest thereby inhibiting the proliferation and invasion of tumor cells [84]. However, PI-103 was halted in the preclinical period due to the poor pharmacokinetic properties. NVP-BEZ235 is a promising PI3K/mTOR dual inhibitor exhibiting improved anti-tumor potential compared to rapamycin analogs [85-88]. In preclinical test, study demonstrated that NVP-BEZ235 significantly prolonged the survival of tumor bearing animals without eliciting obvious toxicity [89]. Therefore, NVP-BEZ235 has entered phase I and phase II clinical trials with everolimus in patients with malignant solid tumors (NCT01508104). Other dual PI3K and mTOR inhibitors, such as PKI-587 and XL-765, have shown favorable activity in preclinical settings. XL-765 has completed the trial in combination with radiotherapy and TMZ for GBM as well as in subjects with recurrent GBM (NCT00704080). PKI-587 and XL-765 have recently completed the phase I clinical trials for the treatment of solid tumors (NCT00940498) and recurrent GBM who are candidates for surgical resection (NCT01240460).

## THE LIMITED FACTORS OF TARGETED THERAPY BASED ON PI3K SIGNALING PATHWAY

Though more and more PI3K/Akt/mTOR targeted drugs emerge, they are still undergoing preclinical or clinical trials. Targeted therapy for GBM has yet to demonstrate an appreciable clinical survival benefit. At present, here are some possible reasons for the limited therapy effect: (1) Blood Brain Barrier. It's the most likely explanation for why targeted drugs cannot reach effective concentrations (2) Heterogeneity of GBM. No doubt the outcome of drug efficacy is much influenced by the genetic background of the tumor. In malignant tumors, molecular phenotype of the same tumor in different location may totally diverse and molecular phenotype of the same tumor in different people may also vary. Thereby the sensitivity to targeted therapy may vary. (3) The activation of alternative pathways leads to immune escape. In clinical trials, only a small proportion of the clinical trials in malignant gliomas concurrently conducted pharmacokinetic studies and most of these studies collect blood samples to work out plasma clearance, rather than directly analysis of cerebrospinal fluid or drug concentration in tumor tissues. Collectively, there are all relevant restrictions to targeted therapy based on PI3K signal pathway.

**Table 3:** Ongoing clinical trials in brain tumors targeting mTOR.

Drug	Targets	Combination Partner	Patient group	Phase	State	Trail ID
Sirolimus	mORC1		GBM	I/II	completed	NCT00047073
		vaccine therapy	NY-ESO-1 expressing solid tumors	I	ongoing	NCT01522820
Sirolimus, Erlotinib	mTORC1 +EGFR		glioblastoma	II	completed	NCT00672243
			malignant glioma	I/II	completed	NCT00509431
Sirolimus, Vandetanib	mTORC1 +VEGF		glioblastoma	I	completed	NCT00821080
Everolimus, Temozolomide	mTORC1		GBM	I	completed	NCT00387400
		radiation	GBM	I/II	ongoing	NCT01062399
		radiation	glioblastoma	I/II	ongoing,	NCT00553150
Everolimus, Gefitinib	mTORC1+ EGFR		progressive GBM	I/II	completed	NCT00085566
Everolimus, Gleevec, Hydroxyurea	mTORC1, PDGFR BCR-AbI			I	completed	NCT00613132
Everolimus, Temozolomide Bevacizumab	mTORC1, VEGF	radiation	GBM	II	completed	NCT00805961
Everolimus, AEE788	mTORC1, EGFR, VEGFR		GBM	I/II	completed	NCT00107237
Everolimus, BEZ235	mTOR, PI3K/mTOR		Cancer	I/II	unknown	NCT01508104
Everolimus, Sorafenib	mTORC1, RAF		recurrent high-grade gliomas	I/II	recruiting	NCT01434602
Temsirolimus	mTORC1		brain and central nervous system tumors	I	completed	NCT00784914
			brain and central nervous system tumors	I/II	completed	NCT00022724
			GBM	II	completed	NCT00016328
Temsirolimus, Doxorubicin	mTORC1		resistant solid malignancies	I	completed	NCT00703170
Temsirolimus, Docetaxel	mTORC1		resistant solid malignancies	I	completed	NCT00703625
Temsirolimus, Temozolomide,	mTORC1	radiation	GBM	I	completed	NCT00316849
			glioblastoma	II	ongoing	NCT01019434
Temsirolimus, Sorafenib, Erlotinib, Tipifarnib	mTORC1 +EGFR		recurrent GBM or gliosarcoma	I/II	completed	NCT00335764
Temsirolimus, Erlotinib,	mTORC1 +EGFR		recurrent malignant glioma	I/II	completed	NCT00112736
Temsirolimus, Perifosine	mTORC1, +Akt		malignant gliomas	I/II	ongoing	NCT01051557
		cytoreductive surgery, Immuno-suppressant	brain tumor	II	recruiting	NCT02238496
Temsirolimus, Bevacizumab	mTORC1 +VEGF		GBM	II	completed	NCT00800917
Ridaforolimus	mTOR		Glioma	I	completed	NCT00087451
CC-115	DNA-PK/mTOR		advanced solid tumor	I/II	ongoing	NCT01353625
CC-223	dual mTOR inhibitor	surgery, supportive care	advanced solid tumor	I/II	ongoing	NCT01177397
XL765, Temozolomide	dual PI3K/mTOR	radiation	GBM	I	completed	NCT00704080
XL147, XL765	PI3K, PI3K/mTOR		glioblastoma, astrocytoma, Grade IV	I	completed	NCT01240460
PKI-587	PI3K, class IA, mTORC1/C2		solid tumor	I	completed	NCT00940498
AZD8055	mTOR		GBM	I	completed	NCT01316809
INK128	mTORC1/2	Bevacizumab	recurrent glioblastoma, advanced solid tumors	I	recruiting	NCT02142803
Pembrolizumab, Pictilisib, NVP-BEZ235, Ipatasertib	PI3Kα/δ, PI3K/mTOR, Akt1/2/3		Glioblastoma	I/II		NCT02430363
Metformin Temozolomide	mTOR		GBM	I	ongoing	NCT01430351
Metformin	mTOR	radiation	recurrent brain tumor	I	recruiting	NCT02149459

Clinical data related to the EGFR was searched until Nov, 2015.

## CONCLUSION AND FUTURE PROSPECTS

As we have discussed here, PI3K/Akt/mTOR signal pathway after activation of EGFR is one of the most significant signal pathways in tumor cells. It has confirmed that it plays an important role in the genesis and development of glioma. At the moment, targeted therapy towards intracellular signal pathways has not achieved satisfactory result yet. A future perspective for GBM therapy is combination of multiple targets and personalized treatment. Although issues like cross-talk signal pathways or tumor heterogeneity tarnished the efficacy of therapy targeted PI3K/Akt/mTOR as we expected, we still believe that it will light up a new way in glioblastoma therapy. Recent study showed that targeting HSP90 and histone deacetylases could enhance the therapeutic effect of TMZ combined with radiotherapy [90].

## Abbreviations

GBM: glioblastoma multiform
PI3K: phosphatidylinositol 3-kinase
mTOR: rapamycin-sensitive mTOR-complex
EGFR: epidermal growth factor receptor
TMZ: temozolomide
RTKs: receptor tyrosine kinases
PTEN: phosphatase and tensin homolog deleted on chromosome ten
pS6k: ribosomal protein S6 kinase
eIF4E: eukaryotic initiation factor 4E
4EBP1: eukaryotic initiation factor binding protein 1
PIP2: phosphatidy-linositol-3, 4-bisphosphate
PIP3: phosphatidy linositol-3, 4, 5-bisphosphate
BBB: blood-brain-barrier
PDK1/2: phosphoinositide-dependent kinase 1/2
